# Physiological Impact of Right Gastric Artery Ligation During SADI-S: A Prospective Randomized Exploratory Study

**DOI:** 10.1007/s11695-026-08717-y

**Published:** 2026-05-21

**Authors:** Ana Marta Pereira, Sofia S. Pereira, Sofia B. Oliveira, Madalena Santos, José Manuel Oliveira, Tiago Vieira, Mário Nora, Mariana P. Monteiro, Marta Guimaraes

**Affiliations:** 1 Department of General Surgery, Unidade Local de Saude de Entre Douro e Vouga, Santa Maria da Feira, Portugal; 2 Unit for Multidisciplinary Research In Biomedicine, School of Medicine and Biomedical Sciences, University of Porto, Porto, Portugal; 3 ITR - Laboratory for Integrative and Translational Research in Population Health, Porto, Portugal; 4 Atrys Medicina Molecular, Porto, Portugal

## Abstract

**Introduction:**

Single-anastomosis duodenoileal bypass with sleeve gastrectomy (SADI-S) was developed as a simplified alternative to biliopancreatic diversion with duodenal switch but remains technically demanding. Ligation of the right gastric artery (RGA) may facilitate duodenal mobilization and reduce anastomotic tension; however, concerns persist regarding anastomotic perfusion and vagal fiber disruption. This study aimed to assess the safety and physiological effects of RGA ligation, including its potential impact on vagal function, using pancreatic polypeptide (PP) as an indirect marker, as well as gastric and gallbladder emptying.

**Methods:**

In this prospective double-blind randomized exploratory trial conducted at a single public bariatric center, patients undergoing SADI-S were randomized to RGA ligation (*n* = 10) or no ligation (*n* = 9). Participants underwent clinical evaluation, liquid mixed-meal tolerance tests to assess glucose and PP dynamics, gastric emptying scintigraphy, and hepatobiliary scintigraphy before and 12 months after surgery.

**Results:**

Clinical outcomes were comparable between groups, with no differences in postoperative morbidity or mortality. After surgery, both groups exhibited similar rates of symptoms consistent with dumping syndrome during the mixed meal test, along with reduced PP secretion. No correlation was observed between dumping symptoms and PP levels. Gastric emptying and hepatobiliary scintigraphy revealed no significant differences between groups before or after surgery. No symptoms suggestive of biliary reflux were reported.

**Conclusion:**

In this prospective randomized study, no significant differences were observed between groups across the evaluated outcomes. RGA ligation was not associated with adverse clinical or physiological effects compared to no ligation within the limitations of this cohort. However, the small sample size limits statistical power and the ability to exclude clinically meaningful differences; therefore, these findings should be considered exploratory and require confirmation in larger studies.

**Supplementary Information:**

The online version contains supplementary material available at 10.1007/s11695-026-08717-y.

## Key Points

RGA ligation may help facilitate the duodenoileal anastomosis in SADI-S.

RGA ligation was not associated with changes in vascularization or surrogate markers of vagal innervation.

RGA ligation was not associated with different clinical outcomes after SADI-S.

RGA ligation appears feasible during SADI-S in this cohort.

**Supplementary Information:**

The online version contains supplementary material available at 10.1007/s11695-026-08717-y.

## Introduction

Metabolic bariatric surgery (MBS) in patients with grade III obesity or higher remains challenging due to technical difficulties and the high prevalence of obesity-related conditions, which increase surgical risk [1]. Single-anastomosis duodenoileal bypass with sleeve gastrectomy (SADI-S) is a simplified version of biliopancreatic diversion with duodenal switch (BPD/DS) and has been implemented in many centers for this patient population [2].

Omega reconstructions have been adopted in MBS to streamline the procedure [3]. The recognition of the pylorus as a key regulator of physiological gastric emptying and a functional barrier preventing biliary reflux (BR)—thereby reducing the incidence of dumping syndrome and marginal ulcers—has led to the development of pylorus-preserving techniques [4]. BPD/DS arose from a combination of procedures [5, 6], and the post-pyloric anastomosis rendered the SADI-S omega arrangement feasible. Nonetheless, duodenal dissection remains technically demanding, particularly when a short and heavy ileal mesentery must be mobilized to the duodenum, creating tension at the anastomosis [7]. Right gastric artery (RGA) ligation enables duodenal mobilization into a more favorable location, thereby making the process easier and reducing the tension of the anastomosis [7, 8]. Although this technical modification has been reported by some groups and deemed safe, concerns persist regarding impaired anastomotic perfusion [9].

Another concern related to duodenal dissection is pyloric denervation, which may lead to delayed gastric emptying or BR due to pyloric incompetence, particularly when RGA ligation is performed [10–12]. Nonetheless, these complications are uncommon, and the contribution of pyloric dysfunction remains unclear, as pyloric function is not routinely assessed preoperatively and available studies do not distinguish outcomes based on RGA ligation status [13, 14].

Gastric emptying scintigraphy (GES) and hepatobiliary scintigraphy (HIDA) can be used to quantitatively assess gastrointestinal motility and transit, which are regulated in part by vagal innervation [15]. While HIDA is primarily used to assess gallbladder contraction and emptying, it can also indirectly reflect vagal function, as impaired gallbladder emptying on scintigraphy may suggest vagal dysfunction [16]. Beyond motility, vagal signaling plays a central role in gut–brain communication and mediates the effects of several enteroendocrine hormones involved in appetite regulation and glycemic control, including cholecystokinin, glucagon-like peptide-1, peptide YY, and serotonin [17].

Pancreatic polypeptide (PP) secretion is influenced by vagal stimulation and has been proposed as an indirect marker of vagal integrity. PP is released in a biphasic postprandial pattern, with the early phase mediated by vagal activation and the later phase driven by hormonal stimuli [18]. PP has been associated with modulation of gastric emptying, reduces gallbladder contraction, and satiety; thus, postprandial PP levels are considered surrogate markers of vagal and pancreatic islet function, although they may also be influences by metabolic and hormonal changes [19, 20].

To date, whether RGA ligation induces pyloric dysfunction and its related manifestations remains unproven, as no randomized controlled trial has addressed this question. Thus, this study aimed to evaluate the safety of RGA ligation during SADI-S and its potential impact on vagal innervation by analyzing physiological responses to a mixed meal tolerance test (MMTT), including glucose and PP hormone dynamics, dumping-like symptoms, and scintigraphic gastric and gallbladder emptying, before and after surgery. Secondary outcomes included weight loss and the resolution of obesity-related diseases.

## Methodology

This prospective, double-blind randomized trial was conducted at a single public bariatric center as a subanalysis of the SURIDIAB2 study (ClinicalTrials.gov), following Institutional Ethics Committee approval. Adults undergoing SADI-S were randomized (1:1) to right gastric artery (RGA) ligation or no ligation using a computer-generated sequence with concealed allocation (predefined sequence held by independent investigator). Patients, outcome assessors, and analysts were blinded to group assignment, whereas surgeons could not be blinded due to the nature of the intervention. Written informed consent was obtained from all participants, and the study adhered to CONSORT guidelines [21].

### Study Population

**Fig. 1:**
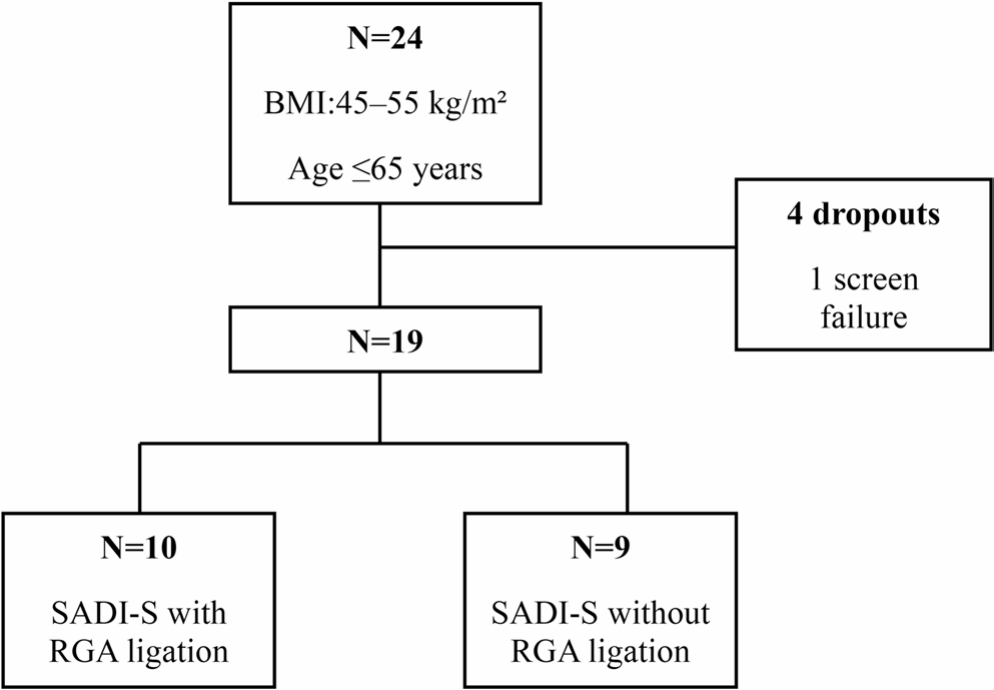
Flowchart of patients enrolled in the study. SADI-S, single anastomosis with duodenoileal bypass and sleeve gastrectomy; RGA, right gastric artery

Twenty-four adults with obesity undergoing SADI-S were enrolled and randomized (1:1) to SADI-S with or without RGA ligation. After four dropouts and one screen failure, 19 patients completed the study and were analyzed (RGA ligation, *n* = 10; no RGA ligation, *n* = 9). Inclusion criteria were body mass index 45–55 kg/m² and age ≤ 65 years. Exclusion criteria included type 2 diabetes, previous gastrointestinal surgery, gastroesophageal reflux disease (GERD) refractory to proton pump inhibitors (PPIs) and confirmed by endoscopy, and BR confirmed by esophageal impedance. All patients underwent preoperative upper gastrointestinal endoscopy. The study flowchart is shown in Fig. 1.

### Study Design

Patients were followed for up to 2 years after surgery, although study-specific assessments were conducted preoperatively and at 3, 6, and 12 months. Assessments included medical history, physical examination and anthropometric measurements to calculate BMI, total weight loss (TWL), and percentage of excess BMI loss (%EBMIL). At each study visit, a MMTT was performed after a 12-hour overnight fast. Participants ingested a standardized liquid meal (Fresubin Energy Drink, 200 mL, 300 kcal [50E% carbohydrate, 15E% protein, and 35E% fat]) within 15 min. Vital signs and symptoms compatible with dumping syndrome were recorded. Venous blood samples were collected at -30 and 0 min before meal ingestion and at 15, 30, 45, 60, 90, and 120 min thereafter. Samples were centrifuged, and the plasma was separated and stored at – 20 °C until further analysis.

GES and HIDA were performed before and 12 months after surgery. For GES, the same standardized liquid meal was labeled with 37–74 MBq of Tc-99 m-DTPA, and planar abdominal images were acquired immediately after ingestion and at 1, 2, 3, and 4 h postprandially. Gastric retention (GR) was calculated using a gastric region of interest defined over the stomach, with the immediate post-ingestion image as reference.

Hepatobiliary scintigraphy was performed after intravenous administration of 185–222 MBq of Tc-99 m mebrofenin. Abdominal images were acquired before the test meal and at 20, 40, and 60 min postprandially. A region of interest (ROI) was drawn around the gallbladder, and gallbladder ejection fraction (GBEF) was calculated as the percentage reduction in gallbladder radioactivity relative to the pre-meal image. As the analysis was restricted to gallbladder counts, tracer activity in the duodenum did not affect GBEF calculation. The same protocol was used preoperatively and at 12 months. Image acquisition and analysis followed established guidelines for GES [22] and HIDA [23] .

### Surgical Procedures

SADI-S consisted of sleeve gastrectomy (SG) followed by a duodenal–ileal anastomosis. SG was performed using a 36-French bougie, with gastric transection starting 4 cm proximal to the pylorus. The duodenum was dissected posteriorly, sectioned 2–3 cm distal to the pylorus, and an end-to-side, hand-sewn three-layer duodenal–ileal anastomosis was created, forming a 300-cm common limb measured to the ileocecal valve. In the RGA ligation group, the RGA was ligated at its origin, preserving the lesser curvature arterial arcade. Petersen’s defect was closed. All procedures were performed laparoscopically by the same surgical team.

### Outcome Measures

GES outcomes were expressed as percentages (%) of GR and gallbladder emptying following a standardized mixed meal.

During the MMTT, dumping-related symptoms were assessed using the Sigstad score, with ≥ 4 considered indicative [24]. As the score does not distinguish early dumping from post-bariatric hypoglycemia, symptom timing and glucose response were considered. Symptoms occurring within 30 min with a rise in plasma glucose were classified as early dumping [24]. Heart rate, blood pressure, and plasma glucose were recorded and compared between groups. Patients were also questioned about any symptoms of GERD and BR (reflux symptoms irresponsive to PPIs, biliary vomits).

Blood samples were collected in EDTA tubes, centrifuged to obtain plasma, and stored at − 80 °C until analysis. PP levels were measured using a commercial ELISA kit (ab288177, Abcam, Cambridge, UK) according to the manufacturer’s instructions. Plasma samples were diluted 1:2 in sample diluent prior to analysis. The assay detection range was 6.25–400 pg/mL and an intra-assay coefficient of variation (CV) of 9.4%. All samples were measured in duplicate, and the mean value was used for analysis.

Early (< 90 days) and late morbidity (≥ 90 days) were classified using Clavien-Dindo [25], with late morbidity defined as complications requiring hospitalization or reintervention.

Weight outcomes included BMI, %TWL, and %EBMIL. Resolution of obesity related health problems included hypertension, dyslipidemia, with outcomes reported per Brethauer et al. [26].

### Statistical Analysis

Categorical variables are presented as counts and percentages, and continuous variables as mean ± standard error unless otherwise specified. Group comparisons for categorical data were performed using Fisher’s exact test and effect sizes were expressed as odds ratios (OR) with corresponding 95% confidence intervals (CI). Normality was assessed with the Kolmogorov–Smirnov test. Continuous variables were compared using Student’s t-test or Mann–Whitney U test, as appropriate. Effect sizes were reported as eta-squared (η²), and results were expressed as mean differences with 95% CI.

Total area under the curve (tAUC) was calculated using the trapezoidal rule. MMTT responses were further analyzed using a linear mixed-effects model to account for repeated measurements within individuals. The model included group (RGA ligation vs. no ligation), visit (preoperative vs. 12 months), and MMTT sampling time (0–120 min) as fixed effects. To accommodate the repeated-measures structure, a combined timepoint variable (visit × MMTT time) was used. Similarly, to evaluate differences according to dumping status, MMTT responses were also analyzed using a linear mixed-effects model including group (dumping vs. no dumping), MMTT sampling time (0–120 min), and their interaction as fixed effects, with participant included as a random effect to account for within-subject correlation Effect estimates with 95% CI were reported. Statistical significance was set at *p* < 0.05. Analyses were conducted using GraphPad Prism software version 10.4.1 (GraphPad Software, La Jolla, USA).

## Results

Out of 19 selected patients, 10 underwent SADI-S with RGA ligation, and 9 underwent the procedure without RGA ligation. The preoperative BMI was comparable between the groups (48.28 ± 0.77 and 47.89 ± 0.49, respectively, *p* = 0.680), as well as general baseline characteristics, as illustrated in Table 1. Follow-up rate was 100% in all cases, except for one time-point for one patient in the cohort without RGA ligation who failed to attend the 3-month follow-up visit.

**Table 1:** Preoperative baseline characteristics

*﻿n*	RGA ligation	No RGA ligation	Mean difference (95% CI)	Effect size	*p*
	10	9	-	-	-
Demographic, anthropometric data					
Age, years	42.70 ± 3.73	39.56 ± 3.45	-3.14 (-13.95–7.66)	0.02	0.547
Sex n. M/F (%)	2/8 (20.0/80.0)	5/4 (55.6: 44.4)	-	5.00 (0.76–31.00)	0.170
Weight, kg	131.60 ± 5.33	136.00 ± 4.66	4.40 (-10.68–19.48)	0.02	0.546
BMI, kg/m^2^	48.28 ± 0.77	47.89 ± 0.49	0.39 (-2.36–1.58)	0.01	0.680
EBMI, %	102.50 ± 3.35	100.67 ± 2.14	-1.79 (-10.40–6.81)	0.01	0.666
Comorbidities, %					
DL	3 (33.3)	5 (55.6)	-	1.56 (0.27–11.22)	0.637
HT	9 (90.0)	8 (88.9)	-	1.13 (0.05–23.51)	1.000
Sleep Apnea	1 (10.0)	1 (11.1)	-	0.89 (0.04–18.81)	1.000
OA	4 (40.0)	4 (44.4)	-	0.83 (0.14–4.92)	1.000
Depression	3 (30.0)	2 (22.2)	-	1.50 (0.24–10.28)	1.000

*BMI* body mass index, *DL* dyslipidemia, *EBMI* excess body mass index, *HT* hypertension, *MS* metabolic syndrome, *M/F* male/female, *OA* osteoarthritis, *RGA* right artery ligation

No statistically significant differences were found between groups (p>0.05). For continuous variables, effect sizes are reported as eta-squared (η²), whereas for categorical variables effect sizes are expressed as odds ratios (OR) with corresponding 95% confidence intervals (CI)

### Gastric Emptying and Hepatobiliary Scintigraphy

**Fig. 2:**
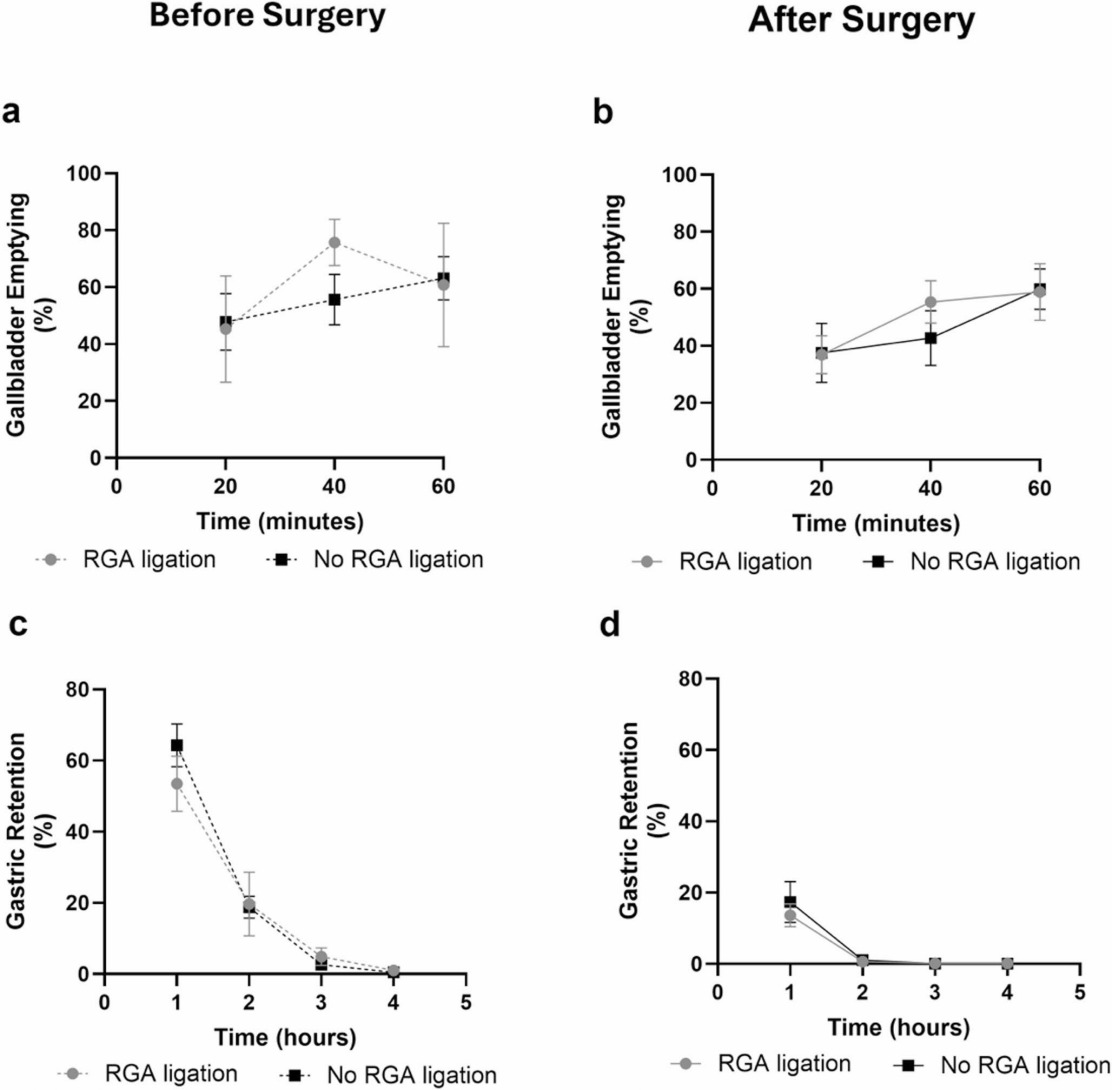
Gallbladder emptying (**A **and **B**) and gastric retention (**C **and **D**) in patients undergoing single anastomosis duodenoileal bypass with sleeve gastrectomy (SADI-S) with or without right gastric artery (RGA) ligation, assessed before (**A **and **C**) and 12 months after surgery (**B **and **D**) using hepatobiliary scintigraphy and gastric emptying studies No significant differences were observed between groups (p > 0.05)

Preoperative GES was similar between groups. One-hour GR markedly decreased post-surgery in both groups, approaching 0% after two hours versus four hours preoperatively, with no significant intergroup differences. Preoperative HIDA scans one-hour post-meal showed normal GBEF. Post-surgery, the radiotracer was detected before the meal, with no significant postprandial change.In the RGA ligation group, mean GBEF decreased from 45.3 ± 18.7% before surgery to 36.9 ± 6.7% after surgery at 20 min, from 75.7 ± 8.1% to 55.4 ± 7.4% at 40 min, and from 60.8 ± 21.7% to 58.9 ± 10.0% at 60 min. The corresponding mean differences (before–after) were 8.36 (95% CI − 50.09 to 66.81), 20.29 (95% CI − 10.58 to 51.16), and 1.86 (95% CI − 66.53 to 70.25), respectively. In the no RGA ligation group, mean GBEF decreased from 47.8 ± 10.0% before surgery to 37.5 ± 10.4% after surgery at 20 min, from 55.6 ± 8.8% to 42.7 ± 9.6% at 40 min, and from 63.1 ± 7.6% to 59.8 ± 7.1% at 60 min. The corresponding mean differences (before–after) were 10.28 (95% CI − 29.49 to 50.05), 12.96 (95% CI − 23.61 to 49.53), and 3.28 (95% CI − 25.34 to 31.89), respectively. No differences were observed between groups (Fig. 2).

### Clinical Response During the MMTT

**Fig. 3:**
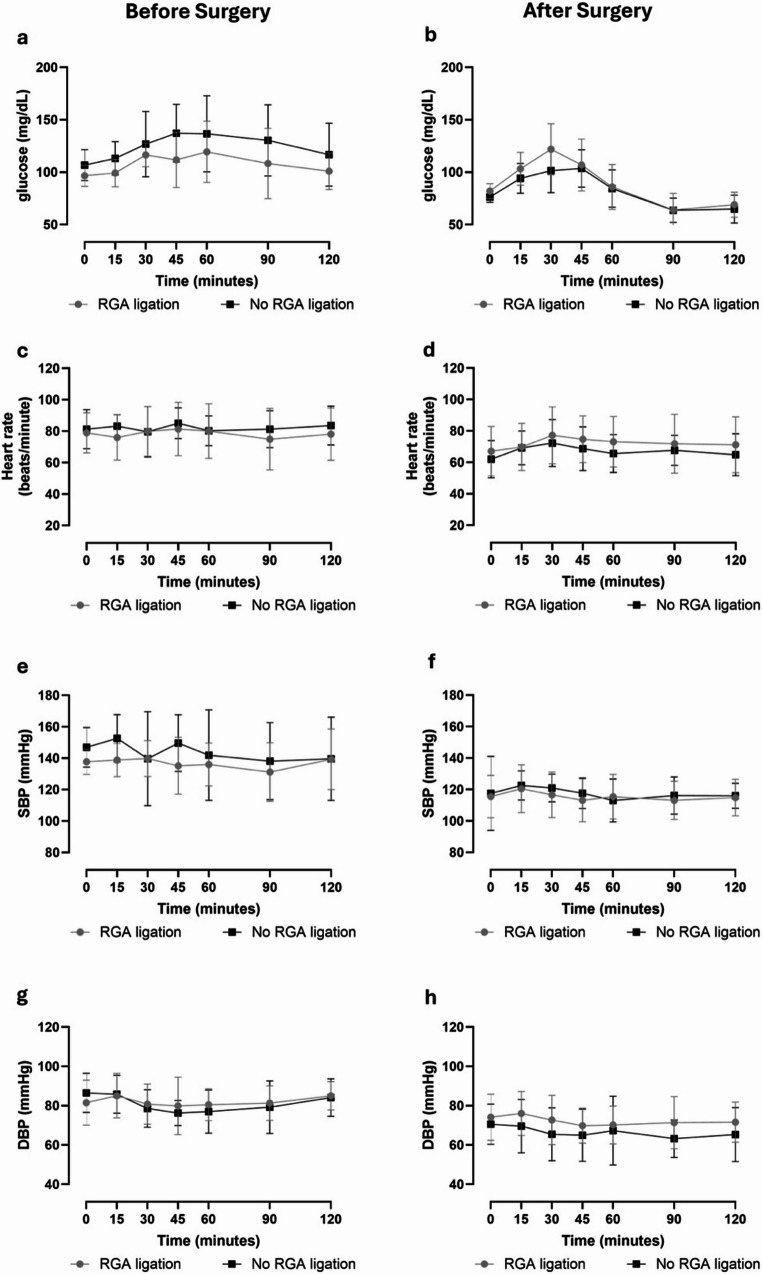
Glucose excursion (**A **and **B**) and vital parameters, including heart rate (**C **and **D**), systolic blood pressure (SBP; **E** and **F**), and diastolic blood pressure (DBP; **G **and **H**), during the mixed meal tolerance test (MMTT) before surgery (**A**, **C**, **E**, **G**) and 12 months postoperatively (**B**, **D**, **F**, **H**) in patients with an without right artery (RGA) ligation. No significant differences were observed between groups (p > 0.05)

During the 12-month MMTT, five patients (three in the RGA ligation group and two in the non-ligation group) experienced dumping symptoms. No significant differences were seen between the groups. SBP and DBP variation, HR, and glucose excursion during MMTT were similar for both groups at all timepoints, as depicted in Fig. 3. Two participants in each group reported mild symptoms compatible with early dumping syndrome during outpatient visits, when specifically questioned about these symptoms, independent of the MMTT, which were addressed through dietary adjustments.

### PP Assays

**Fig. 4:**
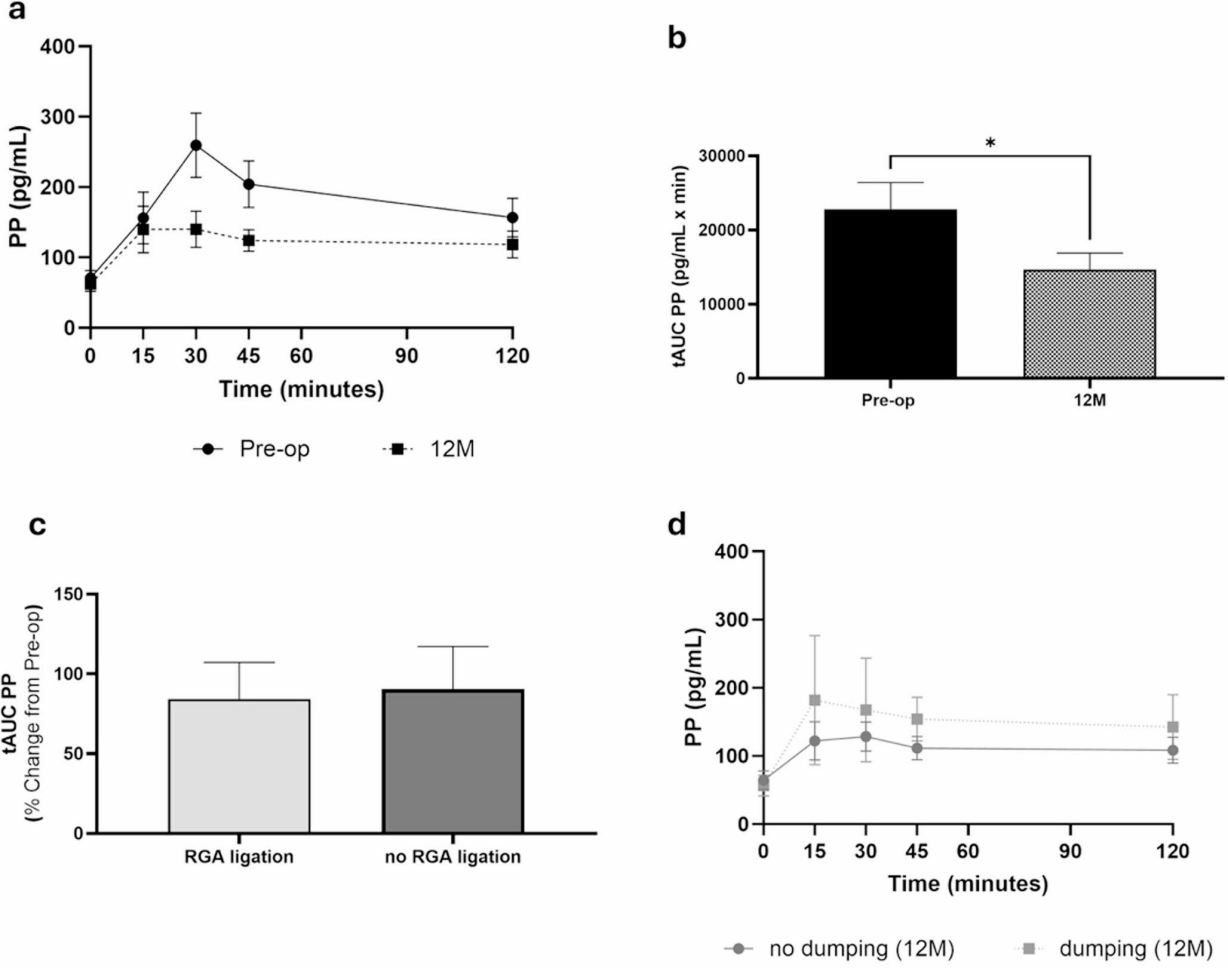
Pancreatic polypeptide (PP) responses during the mixed meal tolerance test (MMTT) before and 12 months after surgery for all patients (**A**) and total area under the curve (tAUC) before and 12 months after surgery for all patients (**B**). Percentage change in PP tAUC from baseline to 12 months in patients with and without right gastric artery (RGA) ligation (**C**). PP responses during the MMTT at 12 months for all patients, stratified by presence or absence of dumping symptoms during the meal (**D**)

PP levels before surgery did not depict significant differences between the groups. In contrast, 12 months after surgery, although fasting levels were similar to those observed before surgery, there was a significantly lower increase during the MMTT (tAUC: 22841 ± 3571 vs. 14699 ± 2179 pg/mL × min, *p* = 0.046) (Fig. 4. A and B). In a mixed-effects model accounting for repeated MMTT measurements (group × visit × time), there was no overall difference between patients with and without RGA ligation (estimated mean difference − 6.8 pg/mL, 95% CI − 98.1 to 84.5; *p* = 0.591) and no significant group × timepoint interaction (F = 1.63, *p* = 0.197), indicating that the PP response profile during the MMTT did not differ between patients with and without RGA ligation. Consistent with these findings, the relative change in tAUC from preoperative to 12-month evaluation was similar between groups (Fig. 4C), indicating that the magnitude of the postoperative decrease in PP response during the MMTT was comparable between patients with and without RGA ligation. The relative change in early PP response, assessed by tAUC 0–30 min (representing the vagally mediated phase), was also evaluated and showed no differences between groups (RGA ligation: 38.77 ± 29.87% vs. no RGA ligation: 26.14 ± 17.77%, *p* = 0.315). PP responses according to dumping syndrome during the test, at 12 M, were also analyzed using a linear mixed-effects model including group (dumping vs. no dumping), timepoint (0–120 min), and their interaction. There was no overall difference between patients with and without dumping (estimated mean difference − 34.0 pg/mL, 95% CI − 134.1 to 66.1; *p* = 0.492) and no significant group × timepoint interaction (F = 0.78, *p* = 0.544), indicating that the PP response during the MMTT did not differ significantly according to the presence or absence of dumping symptoms (Fig. 4. D).

### Safety

RGA ligation did not affect early or late morbidity, which was comparable between groups. No mortality occurred. Operative time, length of hospital stay, and conversion rates were similar.

In the RGA ligation group, one patient underwent reoperation one year later for chronic abdominal pain due to a suspected internal hernia, which was not confirmed laparoscopically. In the non-ligation group, one patient required urgent laparotomic surgery one month postoperatively for segmental small bowel ischemia from superior mesenteric vein thrombosis. Complications are summarized in Table 2.

One patient without RGA ligation had preoperative GERD that resolved after surgery. During follow-up, GERD symptoms occurred in two patients with and three without RGA ligation and resolved with PPIs; importantly, no symptoms of BR were observed.

**Table 2:** Postoperative complications

Mortality	RGA ligation	No RGA ligation	*p*
	0 (0.0%)	0 (0.0%)	1.000
Early morbidity (< 90 days)	0 (0.0%)	1 (11.1%)	0.474
Late morbidity (≥ 90 days)	2 (20.0%)	0 (0.0%)	0.474
Claviend Dindo (< 90 days)	1 (10.0%)	2 (22.2%)	1.000
Grade I	0 (0.0%)	1(11.1%)	
Grade IIIb	1(10.0%)	0 (0.0%)	
Grade IVa	0 (0.0%)	1(11.1%)	
Complications			
Renal lithiasis	1 (10.0%)	0 (0.0%)	1.000
Chronic abdominal pain	1(10.0%)	0 (0.0%)	1.000
SMV thrombosis	0 (0.0%)	1 (11.1%)	0.474
Reintervention	1 (10.0%)	1 (11.1%)	1.000
Surgery duration (min)	122.1 ± 6.34	119.4 ± 10.33	0.758
Hospital stay (days)	2.3 ± 0.21	2.1 ± 0.11	0.861

No statistically significant differences were found between groups (p>0.05) RGA – right gastric artery; SMV – superior mesenteric vein

Weight loss and resolution of obesity-related diseases

Weight loss represented by BMI, %TWL, and %EBMIL was identical for both groups, as illustrated in Supplementary Fig. 1, with a success rate (TWL > 20%) of 100% at one year (Supplementary Table 1). Obesity-related diseases are represented in Supplementary Table 2. Pre-existing asymptomatic gallbladder lithiasis was present in four patients in the RGA ligation group and one in the non-ligation group, with no postoperative gallstone-related complications.

## Discussion

RGA ligation facilitates SADI-S by reducing tension at the duodenoileal anastomosis, though concerns remain about effects on anastomotic perfusion and vagal innervation. In this study, RGA ligation was not associated with increased morbidity or mortality, and clinical outcomes were similar between groups. During the MMTT, both groups had comparable dumping-like symptoms and reductions in PP, with no association between symptoms and PP response. One year postoperatively, GR decreased and GBEF remained unchanged, with no symptoms of biliary reflux.

The vascular supply of the duodenoileal anastomosis is primarily provided by the lesser curvature arterial arcade and the gastroduodenal artery. When the RGA is ligated at its origin, collateral perfusion through the left gastric artery is preserved, which appears sufficient to maintain gastric submucosal blood flow [27]. Consistent with this anatomical consideration, no ischemic or anastomotic complications attributable to impaired vascularization were observed in our cohort. Notably, only a single case of gastric ischemia related to RGA ligation has been reported in the literature [9].

Accelerated gastric emptying is an expected consequence of the SG component of SADI-S. Increased intraluminal pressure, loss of the gastric pacemaker region, and reduced gastric accommodation all contribute to this effect. Preservation of part of the antrum maintains contractile function and further promotes rapid emptying, even in the presence of an intact pylorus [28, 29]. In this study, significant reductions in GR were observed one year after surgery for both groups, suggesting that RGA ligation did not affect gastric emptying, which appears to be predominantly driven by the SG component.

Disruption of the distal branches of the vagus nerve during SG is less likely when an adequate portion of the antrum is preserved [30]. In contrast, duodenal dissection during SADI-S may compromise terminal branches of the vagus nerve, particularly those supplying the pylorus, liver, and pancreas. Such injury could theoretically impair pyloric relaxation, gallbladder contraction, and pancreatic secretion [31]. Although delayed gastric emptying would be expected due to reduced pyloric relaxation, vagal denervation simultaneously impairs gastric accommodation and enhances antral filling, thereby accelerating emptying. Moreover, duodenal feedback mechanisms that normally delay gastric emptying are also lost, which further contributes to this dysregulation, collectively predisposing to dumping-like symptoms [32]. In addition, complex interactions between gastrointestinal hormones, including pancreatic polypeptide and glucagon, have been implicated in post-bariatric metabolic responses, highlighting the multifactorial regulation of postprandial physiology after bariatric surgery [33].

The reduced PP response observed after surgery in both groups supports the presence of some degree of vagal efferent disruption, likely involving hepatic or distal gastric branches. As PP secretion is influenced by vagal stimulation, diminished postprandial levels may reflect altered vagal signaling following SADI-S. However, the absence of group differences indicates that RGA ligation does not exacerbate this effect.

In addition to neural mechanisms, the anatomical rearrangement inherent to the SADI-S omega configuration alters enteroendocrine signaling. Nutrient bypass of the proximal intestine reduces stimulation of cholecystokinin-secreting cells, potentially impairing gallbladder contraction and modulating gastric emptying through vagal paracrine pathways. Reduced proximal stimulation may therefore contribute to the observed acceleration of gastric emptying and blunted PP secretion, independent of RGA ligation [34].

This study has several limitations, most notably the small sample size, which limits statistical power to detect rare or subtle differences in hormonal and functional endpoints and increases the risk of type II error. Therefore, while no differences were detected between groups, these findings should be interpreted with caution and cannot be considered evidence of equivalence. Second, the follow-up period was relatively short. Third, pancreatic polypeptide was used as an indirect marker of vagal function rather than a direct measure of vagal integrity, and its secretion may be influenced by multiple metabolic and hormonal factors following bariatric surgery. Finally, the use of a liquid test meal for both the MMTT and GES may not fully reflect patients’ usual eating patterns. Nevertheless, this study provides prospective randomized data in an area where high-quality physiological evidence is limited and may serve as a foundation for future larger studies. Further research is warranted to clarify the long-term physiological implications of vagal alterations associated with SADI-S and to help optimize surgical technique.

## Conclusion

In this prospective randomized study, no significant differences were observed between groups across the evaluated outcomes. RGA ligation was not associated with adverse clinical or physiological effects compared to no ligation within the limitations of this cohort. However, the study was underpowered to exclude clinically meaningful differences in rare events; therefore, these findings should be considered exploratory and require confirmation in larger studies.

## Supplementary Information

Below is the link to the supplementary material.

Supplementary File 1 (PNG 354 KB)

High Resolution Image (TIF 213 KB)

Supplementary Material 2

Supplementary Material 3

## Data Availability

The data that support the findings of this study are not openly available due to reasons of sensitivity and are available from the corresponding author upon reasonable request. Data are located in controlled access data storage at Unidade Local de Saude de Entre Douro e Vouga.
