# Supplementary figures and images for: Physiological Impact of Right Gastric Artery Ligation During SADI-S: A Prospective Randomized Exploratory Study
Source: Obes Surg. 2026 May 21;36(7):3486–96. doi: 10.1007/s11695-026-08717-y (PMC13323288; doi:10.1007/s11695-026-08717-y)

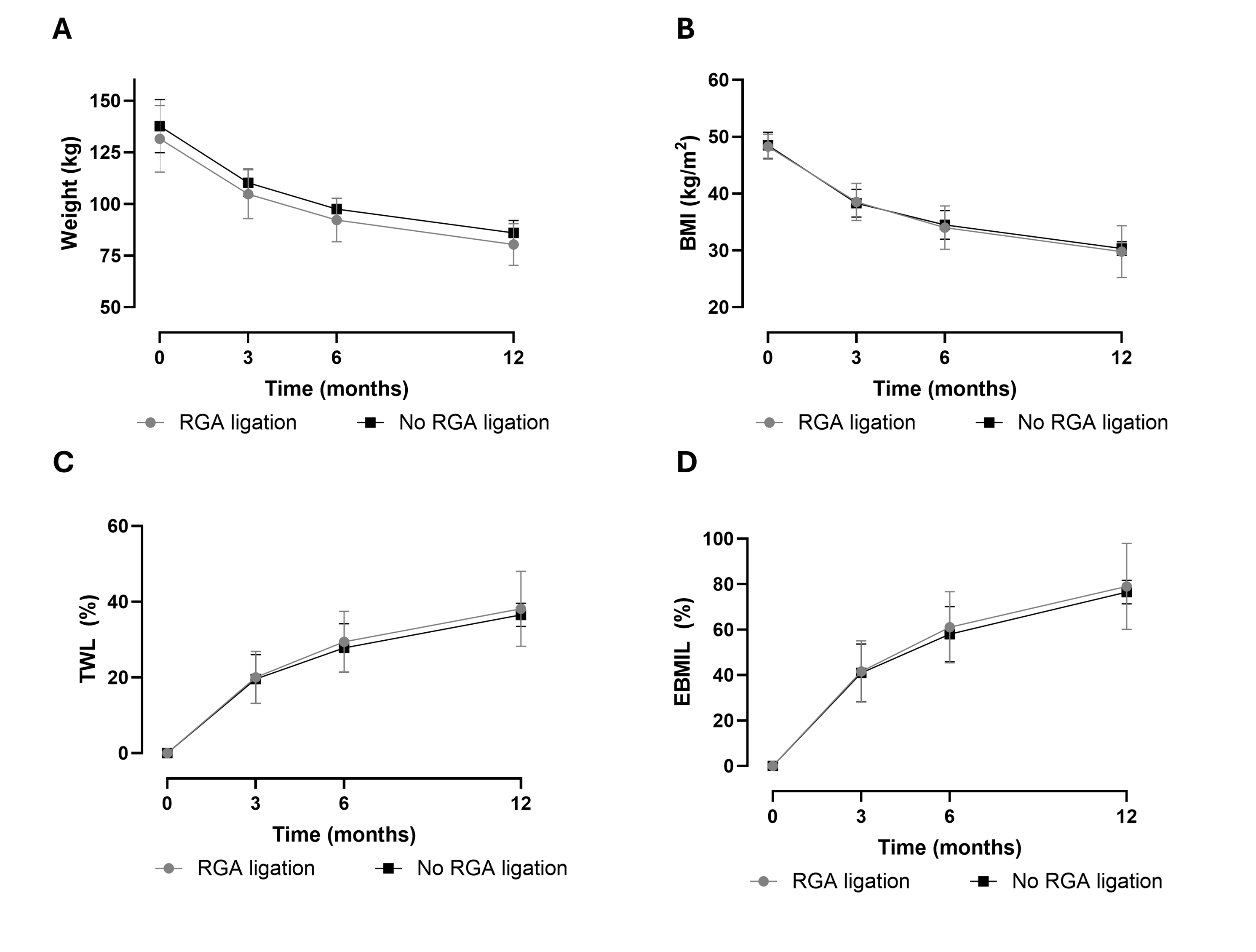

Supplement: Supplementary file 1 — (PNG 354 KB) [file 11695_2026_8717_Fig5_ESM.png]

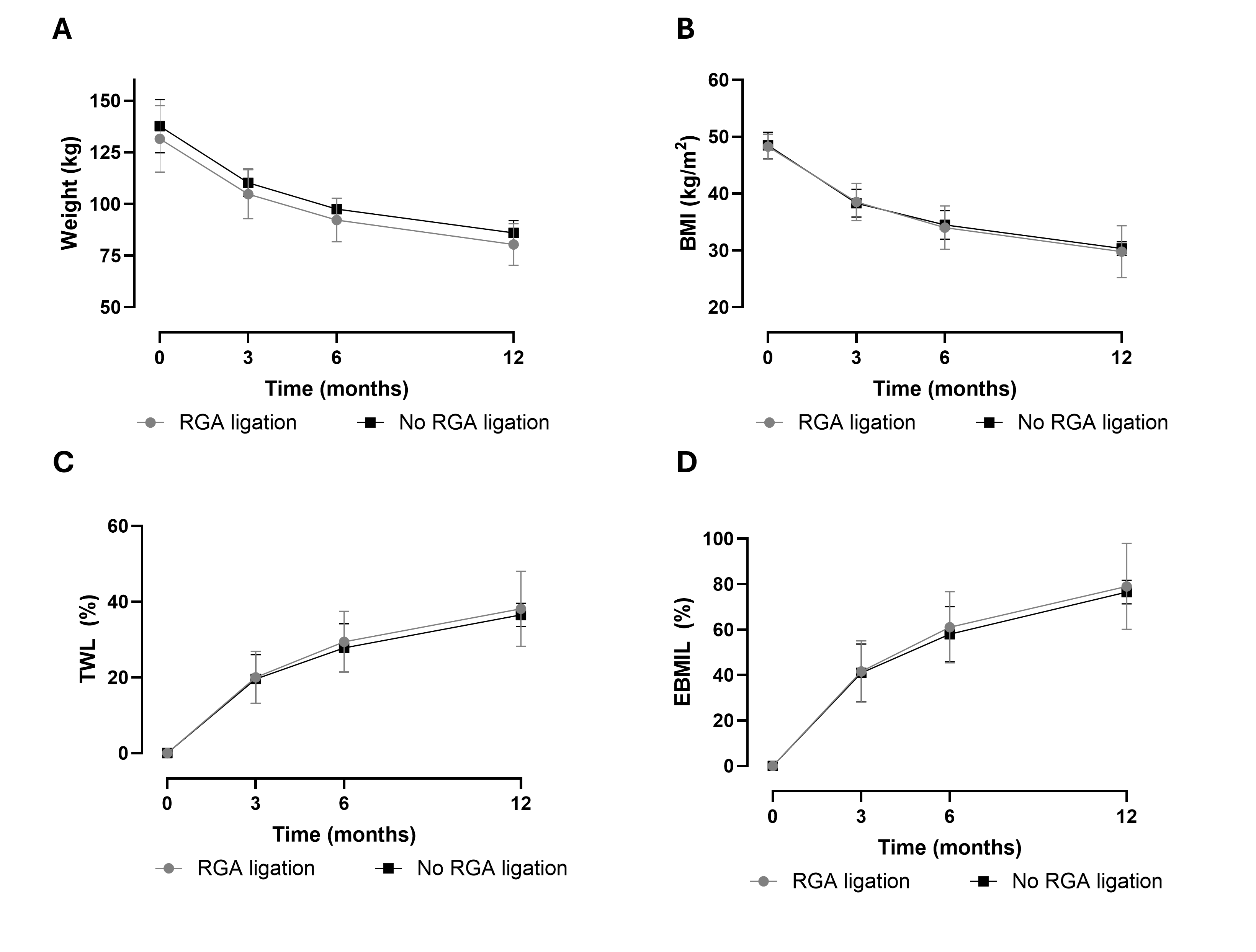

Supplement: Supplementary file 2 — High Resolution Image (TIF 213 KB) [file 11695_2026_8717_MOESM1_ESM.tif]
