# Supplementary material for: Physiological Impact of Right Gastric Artery Ligation During SADI-S: A Prospective Randomized Exploratory Study
Source: Obes Surg. 2026 May 21;36(7):3486–96. doi: 10.1007/s11695-026-08717-y (PMC13323288; doi:10.1007/s11695-026-08717-y)
Supplement: Supplementary file 3 — Supplementary Material 2 [file 11695_2026_8717_MOESM2_ESM.docx]

Supplementary Table 2. Obesity-related disease remission after surgery.

|  | **Preoperative n** | | **Out** | **3 months** | | **6 months** | | **12 months** | |
| --- | --- | --- | --- | --- | --- | --- | --- | --- | --- |
|  | **RGA lig.** | **RGA N-lig.** |  | **RGA lig.** | **RGA N-lig.** | **RGA lig.** | **RGA N-lig.** | **RGA lig.** | **RGA N-lig.** |
| **HT** | 9 | 8 | CR | 4/9(44.4%) | 2/8(25.0%) | 5/9(55.6%) | 4/8(50.0%) | 5/9(55.6%) | 6/8(75.0%) |
| **DL** | 4 | 5 | CR | 0/4(0.0%) | 0/5(0.0%) | 0/4(0.0%) | 0/5(0.0%) | 0/4(0.0%) | 3/5(60.0%) |
|  |  |  | I | 0/4(0.0%) | 0/5(0.0%) | 1/4(25.0%) | 3/5(60.0%) | 2/4(50.0%) | 2/5(40.0%) |
| **OSA** | 1 | 1 | CR | 0/1(0.0%) | 0/1(0.0%) | 0/1(0.0%) | 0/1(0.0%) | 0/1(0.0%) | 0/1(0.0%) |
| **OA** | 4 | 4 | CR | 0/4(0.0%) | 0/3(0.0%) | 0/4(0.0%) | 1/4(25.0%) | 1/4(25.0%) | 1/4(25.0%) |
| **Depression** | 3 | 2 | CR | 1/3(33.3%) | 0/2(0.0%) | 0/3(0.0%) | 0/2(0.0%) | 1/3(33.3%) | 0/2(0.0%) |

No statistical differences were found between groups (p>0.05). Pre-operative “n” represents the number of patients with the respective disease before surgery. Out, outcome; CR, complete remission; I, improved; HT, hypertension; DL, dyslipidemia; OSA, obstructive sleep apnea; OA, osteoarthritis; RGA lig, right gastric artery ligation; SADI-S, single anastomosis with duodenoileal bypass with sleeve gastrectomy; RGA N-lig., no right gastric artery ligation.
