# Supplementary material for: Physiological Impact of Right Gastric Artery Ligation During SADI-S: A Prospective Randomized Exploratory Study
Source: Obes Surg. 2026 May 21;36(7):3486–96. doi: 10.1007/s11695-026-08717-y (PMC13323288; doi:10.1007/s11695-026-08717-y)
Supplement: Supplementary file 4 — Supplementary Material 3 [file 11695_2026_8717_MOESM3_ESM.docx]

|  |  | **RGA ligation** | **No RGA ligation** | **Mean difference (95% CI)** | **p** |
| --- | --- | --- | --- | --- | --- |
| **0** | **BMI, kg/m^2^** | 48.28 ± 0.77 | 47.89 ± 0.49 | −0.24 (-3.13 – 2.64) | 0.999 |
| **3** | **BMI, kg/m^2^** | 38.54 ± 1.03 | 38.31 ± 0.86 | 0.22 (-3.54 – 4.00) | 1.000 |
|  | **EBMIL, %** | 41.54 ± 4.26 | 40.99 ± 4.50 | 0.60 (-16.85 – 18.05) | 1.000 |
|  | **TWL, %** | 20.05 ± 2.18 | 19.60 ± 2.29 | 0.45 (-8.44 – 9.34) | 1.000 |
|  | **TWL≥20%, n (%)** | 4 (40%) | 4 (50%) | - | 1.000 |
| **6** | **BMI, kg/m2** | 34.02 ± 1.21 | 34.51 ± 0.84 | -0.49 (-4.63 – 3.65) | 0.996 |
|  | **EBMIL, %** | 61.07 ± 4.94 | 58.03 ± 4.06 | 3.04 (-14.80 – 20.87) | 0.983 |
|  | **TWL, %** | 29.42 ± 2.55 | 27.80 ± 2.12 | 1.62 (-7.63 – 10.87 | 0.982 |
|  | **TWL≥20%, n (%)** | 9 (90%) | 9 (100%) | - | 1.000 |
| **12** | **BMI, kg/m2** | 29.79 ± 1.44 | 30.37 ± 0.40 | -0.57 (-5.06 – 3.91) | 0.993 |
|  | **EBMIL, %** | 79.02 ± 5.97 | 76.50 ± 1.73 | 2.52 (-16.10 – 21.14) | 0.991 |
|  | **TWL, %** | 38.12 ± 3.14 | 36.54 ± 1.02 | 1.58 (-8.25 – 11.40) | 0.983 |
|  | **TWL≥20%, n (%)** | 10 (100.0%) | 9 (100.0%) | - | 1.000 |

Supplementary material Table 1. Weight loss evolution. BMI, body mass index; EBMIL, excess of BMI loss; TWL, total weight loss; RGA, right gastric artery; CI, confidence intervals (Mixed-effects model): No statistical differences were found between groups (p>0.05)).
